# Trouble in paradise: When two species of conservation and cultural value clash, causing a management conundrum

**DOI:** 10.1002/ece3.10726

**Published:** 2023-11-16

**Authors:** Linda Behrendorff, Rachel King, Benjamin L. Allen

**Affiliations:** ^1^ School of Agriculture and Food Sciences University of Queensland Gatton Queensland Australia; ^2^ Queensland Government Department of Environment and Science Queensland Parks and Wildlife Service K'gari Queensland Australia; ^3^ School of Mathematics, Physics and Computing University of Southern Queensland Toowoomba Queensland Australia; ^4^ Institute for Life Sciences and the Environment University of Southern Queensland Toowoomba Queensland Australia; ^5^ Centre for African Conservation Ecology Nelson Mandela University Port Elizabeth South Africa

**Keywords:** diet, endangered species, Fraser, key threatening process, predation management, threatened species conservation, wildlife management

## Abstract

Threatened species throughout the world are in decline due to various causes. In some cases, predators of conservation or cultural value are causing the decline of threatened prey, presenting a conservation conundrum for managers. We surveyed marine turtle nests on K'gari (formally known as Fraser Island), Australia, to investigate dingo predation of green and loggerhead turtle nests, where each of these species is of conservation value. Our monitoring revealed that 84% of nests were predated by dingoes. Only 16% of nests were not consumed by dingoes, and only 5.7% of nests were confirmed to have successfully hatched. Up to 94% of nests were consumed in some areas, and predation rates were similar across different dingo packs. Information on the available numbers of nests and dingoes in the area indicated that turtle nests alone are sufficient to support extant dingoes over the summer. These results indicate that marine turtle eggs represent a previously unquantified but important food source for dingoes on K'gari, and that turtle nests at this rookery site are under serious threat from dingoes. This research should highlight the importance of prioritising the protection of turtle nests from dingoes or risk losing the entire rookery forever in the near future.

## Graphical abstract

Dingo versus turtles: a management conundrum when species of conservation and cultural significance collide on a World Heritage‐listed island. Implications for conserving both species and the viability of a marine turtle rookery.

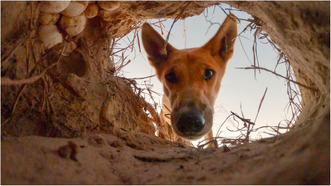

## 1 INTRODUCTION

The global plight of threatened species continues to worsen, and we are now in the midst of a sixth period of mass extinction (Barnosky et al., 2011; Davis et al., 2018). Drivers of this change include land conversion or deforestation, global warming, unsustainable use of natural resources and invasive species, which are all, at least in part, due to increasing human populations (Crist et al., 2017; Lidicker, 2020). Much conservation attention has focussed on the better known and more charismatic species like large terrestrial mammals (Fleming & Bateman, 2016), particularly those in mainland areas where their loss is more easily observed. However, less attention has been given to other taxa and island populations despite their great biodiversity value (Ford et al., 2017). In addition, it can often be difficult for managers to determine the most appropriate course of action in cases where one species of conservation or cultural concern threatens another species of conservation or cultural concern. For example, how should conservation managers respond when an endangered predator threatens an endangered prey? Identifying and understanding these types of conservation conundrums is required to improve conservation actions that can ideally assist all species.

Fraser Island, or K'gari, which means ‘paradise’ to the local indigenous people, or Butchulla [Batjala], is the largest sand island in the world. It is situated a short distance off the south‐east coast of Queensland, Australia, at the very southern end of the Great Barrier Reef. K'gari has rich biodiversity value, including perched freshwater lakes, fens, swamps, old‐growth rainforest growing on sand and a wide variety of terrestrial fauna, including several threatened species such as the long‐nosed potoroo (*Potorous tridactylus*), eastern ground parrot (*Pezoporus wallicus*), black‐breasted button quail (*Turnix melanogaster*) and water mouse (*Xeromys myoides*). Perhaps the most well‐known and iconic wildlife species on the island are dingoes (*Canis familiaris*; Allen et al., 2017; Jackson et al., 2017), which are known to the Butchulla as wongari. Dingoes are protected on K'gari by the Queensland Parks and Wildlife Service (QPWS) for their ecological role on the island and their cultural value to the Butchulla, and the Queensland Government has a legal responsibility to conserve them even though they are a declared pest on the mainland (Queensland Government, 2001). Dingoes can live for over 13 years (Behrendorff & Allen, 2016), and the stable population of 100–200 individuals on the island has great conservation and cultural value (Allen et al., 2015). Dingoes evolved in Asia, arrived in Australia about 3000–5000 years ago and are considered a key component of many terrestrial ecosystems (Fleming et al., 2012), including those on K'gari. Many indigenous people across Australia have a long and shared history and strong affinity for dingoes (Hytten, 2009; Koungoulos, 2021; Rose, 2000; Smith & Litchfield, 2009), including Butchulla people (Carter et al., 2017; Phoenix‐O'Brien, 2002). Contemporary society also highly values dingoes (Van Eeden et al., 2020), and those on K'gari are a major drawcard for over 400,000 tourists visiting the island each year (Thompson et al., 2003; Walker et al., 2022). Dingoes on the island are also a genetically distinct population (Conroy et al., 2017, 2021), and a substantial amount of resources and funding are devoted to conserving and protecting K'gari dingoes (Behrendorff, 2021; Tapply, 2018).

A great diversity of marine fauna is also found in the waters surrounding the island, including several threatened species such as the critically endangered hammerhead shark (*Sphyrna mokarran*), lemon shark (*Negaprion acutidens*), dugong (*Dugong dugon*) and Australian humpback dolphin (*Sousa sahulensis*). Sub‐tidal, soft‐bottom habitats also provide substantial foraging resources for populations of four species of migratory marine turtles, known as milbi to the Butchulla: the loggerhead turtle (*Caretta caretta*), hawksbill turtle (*Eretmochelys imbricata*), flatback turtle (*Natator depressus*) and green turtle (*Chelonia mydas*).

Loggerhead turtles and green turtles have been observed nesting on most of the beaches of the northern half of K'gari at times, although their nesting activity is primarily concentrated on the far northern tip of the island (Strydom, 2004, 2021). Loggerhead turtles were heavily impacted by trawl fishing in the late 20th century before the introduction of Turtle Exclusion Devices (TEDs) in the 1990's, which were made compulsory in 2001 (Limpus, 2008a). Though globally distributed, loggerhead turtle populations in the South Pacific Ocean are dwindling and are presently classified as critically endangered (Limpus & Casale, 2015). Green turtles have been gradually increasing in the region at about 3% per year after all marine turtles became protected species in Queensland in the 1950s (Limpus, 2008b). It is presently estimated that less than 50 loggerhead turtles and an average of 150 green turtles nest on K'gari each year, with over 200 green turtles nesting within the 1996–1997, 1999–2000 and 2002–2003 nesting seasons, the largest being 591 in the 2009–2010 season (Strydom unpub). Green turtles can lay up to eight nests per season (mean 5.06) and loggerhead turtles up to five nests (mean 3.41; Limpus, 1997, 2008a), equating to approximately 1000 turtle nests being laid on K'gari each year. K'gari is therefore considered to be a satellite turtle rookery for these species, and a substantial amount of resources are devoted to conserving and protecting loggerhead turtle nests on the island and other nearby areas. Unfortunately, one of the key threats to marine turtles are dingoes, and several nests are relocated from the original nest site into replicated nests within predator‐proof beach cages situated within the rookery (Strydom, 2004).

Dingoes are predators and scavengers and eat a wide variety of food items, from insects to whales (Behrendorff et al., 2016). Across Australia, their diet mostly consists of terrestrial mammals such as macropods, bandicoots and rabbits (*Oryctolagus cuniculus*; Doherty et al., 2019). However, in specific locations, like K'gari, where their diet has been intensively studied, dingoes have demonstrated remarkable flexibility and capability in their feeding behaviour. Dingoes on the island consume a broad array of food items, including northern brown bandicoots (*Isoodon macrourus*), swamp wallabies (*Wallabia bicolor*) and a range of small rodents (Behrendorff et al., 2016; Twyford, 1995). They also consume large amounts of fish sourced mainly from humans (Déaux et al., 2018), who further supply noteworthy amounts of anthropogenic food and rubbish. Dingoes regularly raid tents, vehicles and storage containers seeking food and causing conflict with human visitors to the island (Allen et al., 2012; Appleby et al., 2018; Behrendorff et al., 2023). Dingoes have also been observed catching difficult‐to‐catch wildlife prey, such as the echidna *Tachyglossus aculeatus*, by using the ocean waves to drown them (Behrendorff, 2018a, 2018b). Almost all of these food items have been recorded from macroscopic features in scat or stomach samples (e.g. hair, bones and scales), but some items do not leave such evidence and have only been observed in person or via camera trapping. These include the many tonnes of meat provided by increasing numbers of stranded whales and other marine mammals (Behrendorff et al., 2018). One of the less frequently observed sources of otherwise undetectable food includes the eggs of loggerhead and green turtles, about which there is very little information (but see Baker, 2005, Haig, 2003 and Strydom, 2004).

Here, we assess the consumption of marine turtle eggs by dingoes on K'gari during the annual marine turtle nesting and Loggerhead Nest Relocation Programme. We used turtle nest monitoring data to quantify the number of turtle nests and eggs laid within the main rookery on the island and the fate of those nests, focussing on dingo predation. Our aim was to assess the importance of this food source for dingoes on K'gari and to better understand the importance of dingo predation on marine turtles.

## 2 METHODS

### 2.1 Animal ethics

Turtle monitoring activities were undertaken following the recommended procedures outlined by the Queensland Department of Environment and Science Threatened Species Unit (Limpus et al., 2006). All activities also received ethical approval from the Department of Agriculture Fisheries and Forestry Animal Ethics Committee (Queensland Turtle Conservation Project: SA 2018‐11‐660, 661, 662, 663, 664).

### 2.2 Study site

**FIGURE 1:**
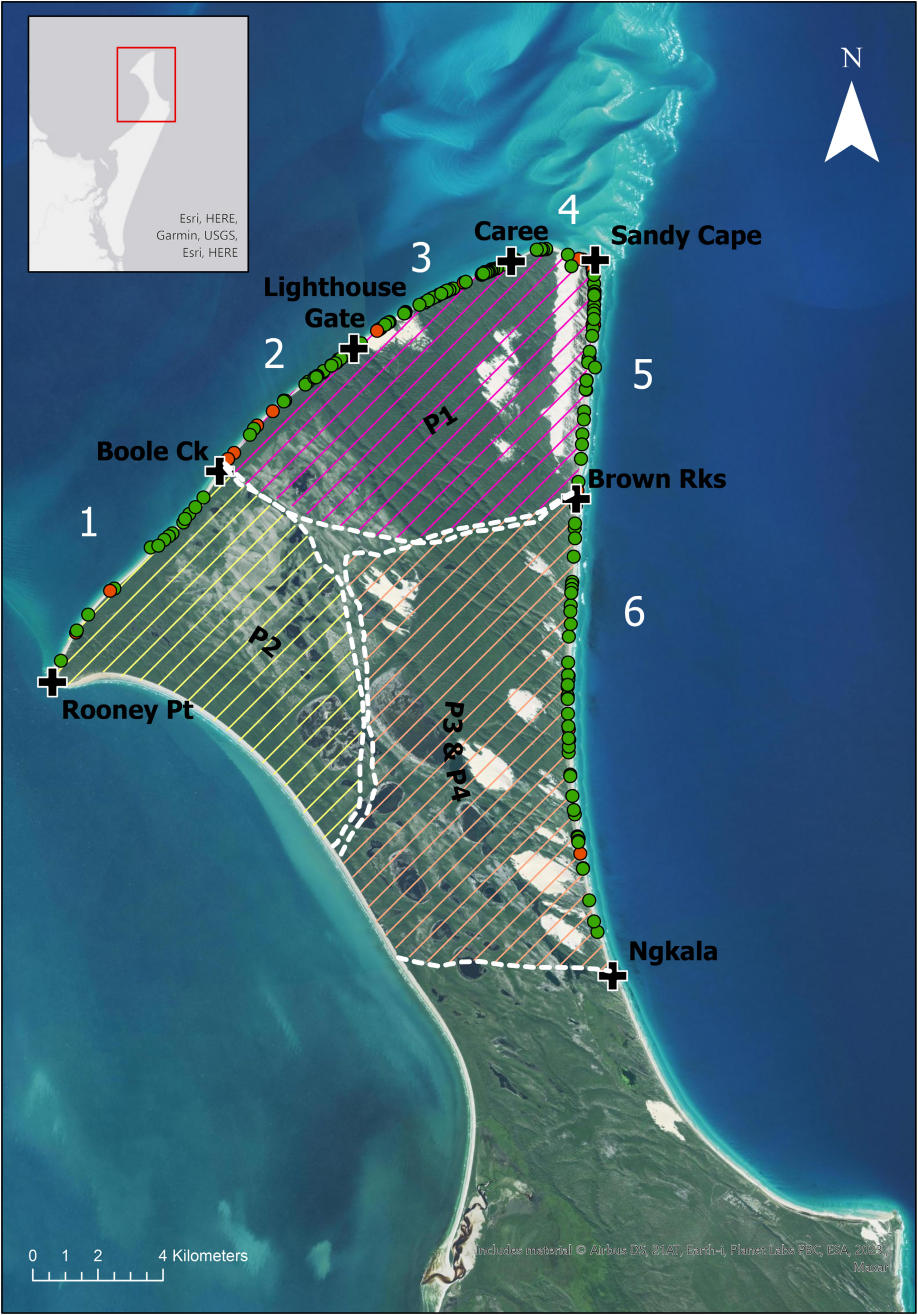
Location of turtle nests (green circles: Green turtle and red circles: Loggerhead) recorded within six survey zones (+ boundary markers), 2019–2021. See Table 1 for zone numbering labels. Lined areas represent the dingo pack territories (P#) assessed (see text for details). The study area is indicated with a red rectangle outline on the inserted map of K'gari.

Turtle nest surveys took place on the northern beaches of K'gari, situated approximately 1.5 km off the south‐east coast of Queensland, Australia, within the Great Sandy National Park (Figure 1). The site has been used for turtle monitoring since 1994 where community volunteers have gathered data on turtle nesting, hatching, emergence and sometimes predation (Strydom, 2004). The park is co‐managed by the QPWS and Butchulla Aboriginal Corporation. Further details on the nature of the study site can be found in Wardell‐Johnson et al. (2015) and Walker et al. (2022).

### 2.3 Turtle nest surveys

Turtle nest surveys occurred over two summer nesting periods between 22 December 2019 and 24 March 2020 in the 2019/2020 nesting season, and between 22 December 2020 and 6 March 2021 in the 2020/2021 nesting season. All beaches from Rooney Point (−24°.81687′, 153°.11755′) to Ngkala Rocks (−24°.89798′, 153°.27213′) were patrolled by vehicle (i.e. approximately 43 km of beaches). This section of island coastline was divided into six zones to facilitate surveying. Survey zones used the same zoning as the historic nesting survey techniques, incorporating landmarks such as rock outcrops to delineate between zones. The turtle nesting area occurred from the intertidal high tide mark up to 100 metres inland. Surveys were conducted daily in each zone where possible during the monitoring period, although there were some days where monitoring did not occur for a variety of reasons. For example, Browns Rocks to Ngkala Rocks (section 6 in Figure 1) could not always be monitored because temporary beach erosion exposed the rocks and made them impassable during part of the 2020/2021 season.

**FIGURE 2:**
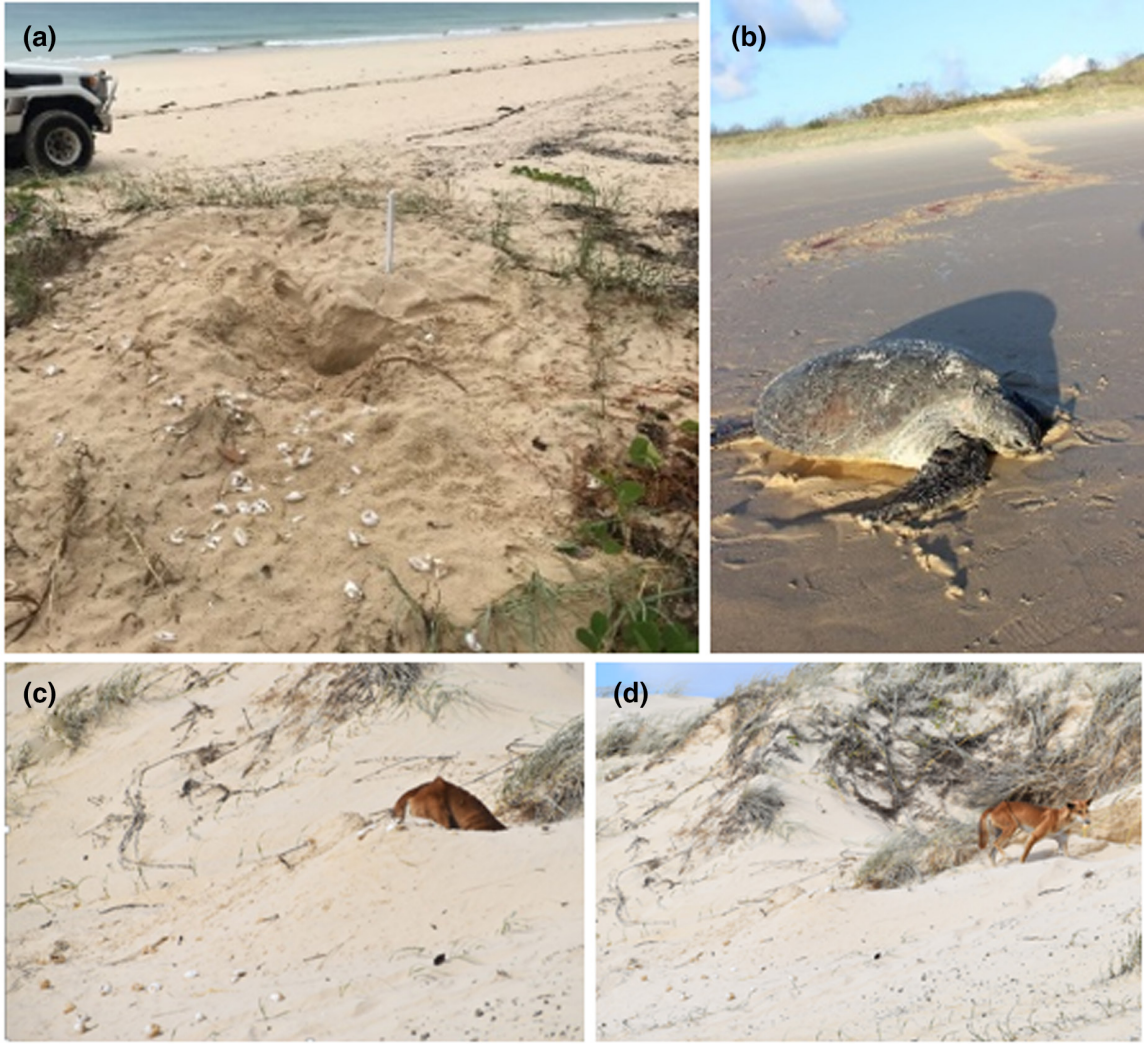
Dingo (wongari) impacts on turtles on K'gari, showing (a) an excavated nest with broken eggshells, (b) an adult turtle attacked on multiple occasions during her return journey to the ocean and (c, d) a wongari excavating a nest and removing eggs. See also Supplementary File.

Nests were located by the obvious species‐specific turtle tracks on the beach (e.g. Figure 2b). All nests laid the previous night that were not already predated, were marked with a white stake, labelled and georeferenced early in the nesting season at the time of first observation. We attempted to mark all loggerhead turtle nests that were not relocated to predator‐proof beach cages, but our marking of green turtle nests only occurred where there were still eggs present (un‐predated), they could be accessed, or if the nest was in thick vegetation. Each marked nest was then checked at relatively frequent but irregular times continuously throughout the remainder of the nesting season, usually daily to every few days. At each check, we recorded the status of the nest (e.g. predated, hatched, relocated and reclaimed by the tide) and the identity of the predator, where relevant (identified from their tracks). Where nests were predated (Figure 1), we estimated the number of eggs consumed by counting the number of broken shells spread around the nest site (Figure 2a below). Where possible, non‐predated nests were excavated after the incubation period of ~64 days (Limpus, 2008b) to assess hatching success. Stakes were removed after the fate of each nest had been determined.

We assessed differences in predation rates between years (nesting seasons) and between the site survey zones or sections of beach we monitored. Because these zones are somewhat arbitrary with respect to dingoes, for additional analyses we further combined them to align with three discrete zones representing the known territories of four local dingo packs (Figure 1).

Due to low rates of non‐predation (Table A1 Supplementary file) across season, monitoring zone and/or pack, a binomial analysis of nest fate (predated/not‐predated) with multiple independent variables was not possible. Analysis of nest fate was therefore limited to chi‐square associations with each individual variable separately so that test assumptions were not violated, that is expected frequencies of all table cells were >1 and at least 80% of the expected frequencies were greater than five (Tabachnick & Fidell, 2012). All chi square tests of association with nest fate (predated/non predated) and tests of proportion were generated using the Real Statistics Resource Pack software (Release 7.6) in Microsoft Excel (Zaiontz, 2020).

Considering only predated nests, the frequency (counts) of predation was modelled using loglinear analysis (Poisson regression) to determine any effect of nesting season (year) and the three pack zones (Figure 1). The 2019 season and the Brown's and Ngkala dingo pack were used as reference levels. This analysis was conducted in R Studio (RStudio Team, 2022) using R software (R Core Team, 2022). The initial six zones of monitoring, turtle species and/or the month that the eggs were laid (November–February) were excluded from this analysis as they resulted in unstable estimates (very large standard errors) or no estimated coefficients due to low sample sizes in variable categories and violation of the model assumptions.

## 3 RESULTS

A total of 201 turtle nests were recorded over both seasons and were collectively checked on 748 occasions. Our sample therefore represented approximately 7%–10% of all the turtle nests laid on the island each year. Of these 201 nests, fates could be confidently determined for only 174 nests, 72 in 2019/2020 representing 41% of our sample and 102 in 2020/2021 representing 59% of our sample. A chi‐square test of independence determined no association between season and nest fate (χ^2^ (1, *N* = 174) = 0.03, *p* = .86), so data from both seasons were pooled for further analyses of nest fate.

Nest checking revealed that 146 (84%; Table 1) nests were predated by dingoes, and no other predators were recorded predating the turtle nests. Of the 28 (16%) nests that were not predated, 10 were collected and relocated, three were washed away during high tides and only 10 (5.7%) were confirmed to have successfully hatched. A further five were assumed to have hatched, though we could not confirm it.

**TABLE 1:** Turtle nest predation rates (%) at the six survey zones or sections of beach monitored on K'gari, 2019–2021 (see Figure 1 for further details).

Location	*N*	Predated	Not predated
1. Rooney Point—Boole Creek^a^	17	0.94	0.06
2. Boole Creek—Lighthouse^a,b^	27	0.78	0.22
3. Lighthouse—Caree Camp Zone^a^	49	0.86	0.14
4. Caree Camp Zone—Sandy Cape^b^	12	0.58	0.42
5. Sandy Cape—Browns Rocks^a,b^	40	0.85	0.15
6. Browns Rocks—Ngkala Rocks^a^	29	0.90	0.10
Total	174	0.84	0.16

*Note*: Superscript letters indicate the results for pairwise tests of predated proportions between locations (*p* < .05).

Predation rates at the six survey zones ranged between 58% in section 5 (Caree Camp Zone to the Lighthouse) and 94% in section 1 (Rooney Point to Boole Creek; Table 1). There was no significant association between the six locations and nest fate (χ^2^ (5, *N* = 174) = 8.74, *p* = .12), indicating that similar proportions of predation and non‐predation occurred at all sites [sample sizes at the six locations ranged from 12 to 49 nests (Table 1)]. Two sample tests of proportion were then used to determine if the proportion of predated nests differed among the six locations. The nests predated between Caree Camp Zone and Sandy Cape (58%, *N* = 12) incurred the lowest proportion of predation of all six locations and were significantly lower than that at the proportions of Rooney Point to Boole Creek (94%, *N* = 17, *z* = 2.34, *p* = .02), Browns Rocks to Ngkala Rocks (90%, *n* = 29, *z* = 2.30, *p* = .02) and Caree Camp Zone to the Lighthouse (86%, *N* = 49, *z* = 2.13, *p* = .03). The proportion of predated nests from Sandy Cape to Browns Rocks (85%, *N* = 40) and Boole Creek to the Lighthouse (78%, *n* = 27) were not significantly different from that from Caree Camp Zone to Sandy Cape, and no other pairwise comparisons identified any significant difference (*p* > .05).

Two sample tests of proportion were also used to determine if the total percentage of predated nests differed among the three dingo pack areas, and again there was no significant difference among the three areas (*p* > .05; Table 2). So, there was no difference between packs when year was ignored. But more nests were predated in 2020 than in 2019, and this difference seemed to be mostly due to increased numbers of nests predated over time by the Rooney's and Sandy Cape packs (Table 3).

**TABLE 2:** Total percentage of nests predated in each by pack area.

Dingo pack	*N*	Predated (%)	Not predated (%)
Rooney's pack (P1)	44	84.1	15.9
Sandy Cape pack (P2)	101	82.2	17.8
Browns and Ngkala packs (P3 and 4)	29	89.7	10.3
Total	174	83.9	16.1

**TABLE 3:** Number of predated nests only, by year and pack zone.

Dingo pack	2019	2020	Total
Rooney's pack (P1)	7	30	37
Sandy Cape pack (P2)	19	64	83
Browns and Ngkala packs (P3 and 4)	14	12	26
Total	40	106	146

Loglinear analysis using only these predated nests (*n* = 146) further showed that there was no difference between years if pack was ignored, but there was an interaction between pack and year in some cases. For example, the odds of predation by the Rooney's pack in 2020 were five times the odds of predation by the Browns and Ngkala packs in 2019 (OR = 5.00, *p* < .01, Table 4), and the odds of predation by the Sandy Cape pack in 2020 were almost four times more likely than predation by the Browns and Ngkala packs in 2019 (OR = 3.93, *p* < .01, Table 4). The Rooney's and Sandy Cape packs appeared to increase the predation of turtle nests in the second turtle nesting season we monitored.

**TABLE 4:** Results of log‐linear analysis showing factors which significantly influence the frequency of predation.

Model	Estimate	SE	*Z*	Pr (>|*z*|)	Odds ratio (95% CI)
Year (2020)	−0.15	0.39	−0.39	0.69	Non‐significant
Rooney's pack	−0.69	0.46	−0.50	0.13	Non‐significant
Sandy Cape pack	0.31	0.35	0.87	0.39	Non‐significant
2020:Rooney's pack	1.61	0.58	2.80	<0 .01	5.00 (1.67, 16.26)
2020:Sandy Cape pack	1.37	0.47	2.90	<0.01	3.93 (1.57, 10.10)

*Note*: The reference levels are year: 2019 and pack: Brown's and Ngkala dingo packs.

**FIGURE 3:**
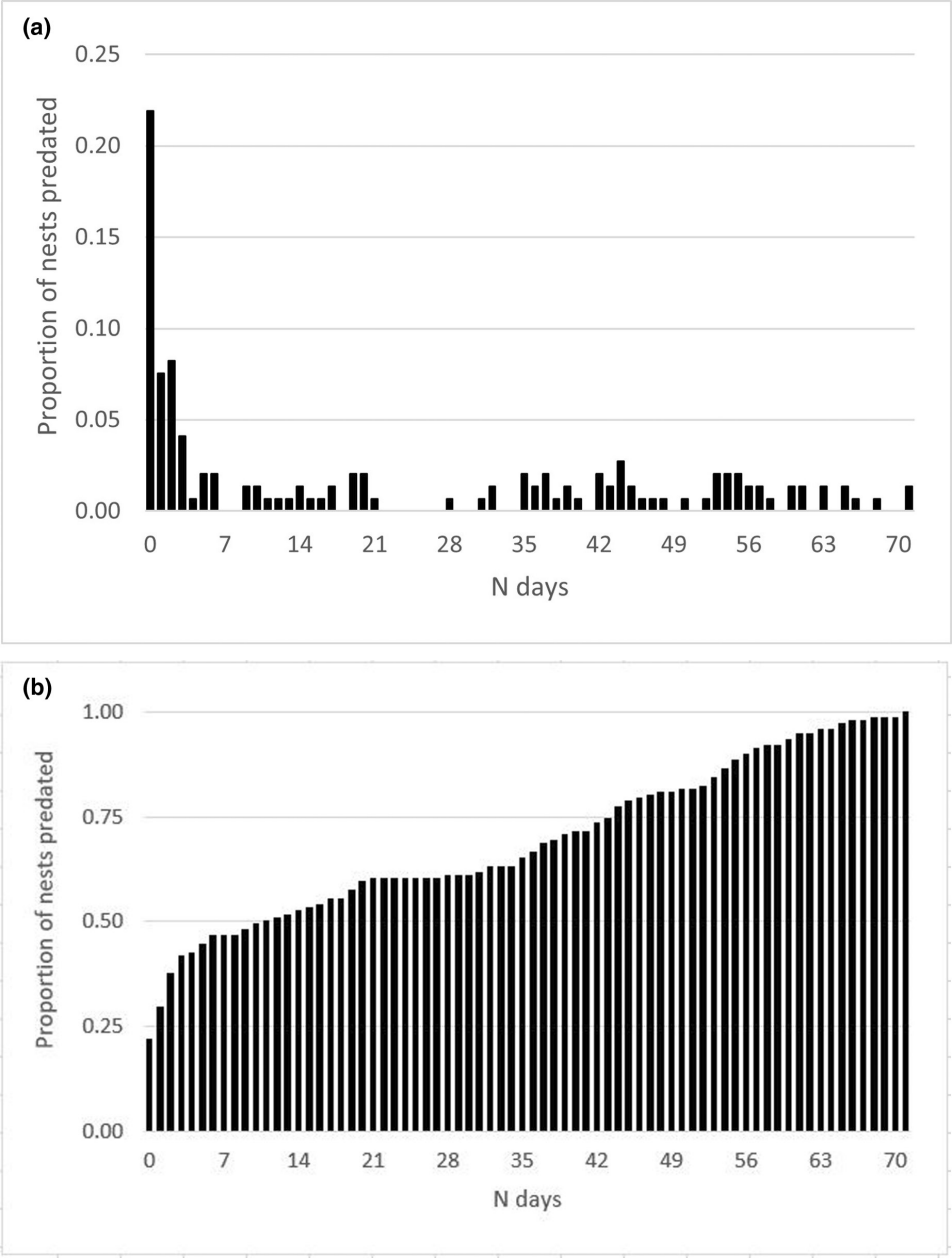
Timing of turtle nest predation by dingoes on K'gari, 2019 to 2021 (a), and cumulative proportion of turtle nests predated by dingoes on K'gari, 2019 to 2021 (b).

A total of 146 of the predated nests had a sufficient number of exhumed and scattered shells for us to estimate how many eggs dingoes consumed (Figure 2). Of these, dingoes consumed a mean of 99.18 (S.E. 2.25, range = 10 to 127) eggs per nest. Only 12 nests belonged to loggerhead turtles, and the rest were from green turtles. Given that most eggs were from green turtles and the mean weight of green turtle eggs is over 40 g (Limpus, 2008b), we estimated that dingoes consumed a mean of 4.97 kg of turtle eggs each time they predated a nest. Predation of turtle nests by dingoes also occurred quickly, with 25% of nests predated on day 1 after laying, 50% by day 11, 75% by day 43 and 100% by day 71 (Figure 3).

## 4 DISCUSSION

The dingo has long been identified as a predator of turtle nests on K'gari (see Supplementary File), but reliable quantitative information on predation rates is scant and difficult to ascertain. From observations of nesting turtles in 1970, Hatch (2015) reported that ‘the dingoes would dig up the sand until they found the right nest and eat them’. Strydom (2004) later reported that ‘dingoes are the major predator of turtle clutches, taking an average of around 50% and up to 86% of nests in some seasons’, that ‘dingoes frequently prey on hatchlings’, and also ‘frequently harass and sometimes kill adult basking and nesting turtles’. The lowest proportion of nests predated by dingoes in that study was 12%–13% in the 2002/2003 nesting season, which Haig (2003) says was derived from a sample of only 23 nests along a 1 km section of beach checked only once, approximately 8 weeks after marking the nests. These reports provided some of the first quantitative data on dingo predation of turtle nests on the island but also highlighted the need to further investigate the issue with more comprehensive sampling. Our study involved repeat sampling of over 200 nests across the entire rookery over two seasons and found similar results (Table 1). This reinforces these earlier findings and suggests that marine turtle nests represent an important food source for dingoes on K'gari and that dingoes could potentially pose a substantial threat to extant marine turtle nests.

Dingoes predated 84% of turtle nests and up to 94% of nests in some areas (Table 1). Very few nests successfully hatched. Four dingo packs occupied the six sections of beach we monitored at the time, which represent three biologically meaningful ‘pack areas’ (Figure 1) collectively containing ~30 dingoes at that time of year (Allen et al., 2015; Baxter & Davies, 2013; White, 2021). Despite some spatiotemporal variation in some places (Table 4), total predation rates during the study period were similar across these three pack areas (Table 2), suggesting that dingoes with access to turtle eggs consume similar proportions of nests. Approximately 200 turtles lay eggs each summer nesting season at the rookery on the northern tip of K'gari, and individual turtles each lay about five clutches in a given nesting season (Limpus, 2008b), meaning that approximately 1000 turtle nests are laid at the rookery each summer. The 84% of nests predated there by dingoes therefore represent the consumption of ~840 nests each year. Dingoes consumed ~100 eggs per nest, equating to ~4 kg of eggs, and dingoes require approximately 1 kg of food per day to meet their energy requirements (Allen & Leung, 2012; Green, 1978). Thus, each turtle nest contains a sufficient number of eggs to provide four dingoes with their daily energy needs. The ~840 nests predated over the ~120‐day nesting period therefore represent approximately seven predated nests per day, on average, or enough food for approximately 28 dingoes during this period. We also observed local dingoes gaining weight over the season and marking nests (urinating on them) before consuming the contents days later (L. Behrendorff, unpublished data). These values are obviously imprecise, and we do not know how many individual dingoes were responsible for the predation of each nest, but they do suggest that turtle nests alone are probably sufficient to support the entire dingo population at the northern end of K'gari over the summer.

Marine turtle eggs therefore represent a previously unquantified but important food source for dingoes on K'gari. The summer timing of this food source also coincides with the dingo dispersal period, when the annual cohort of juveniles becomes independent (Corbett, 2001). At this time, particularly on an island, some dingoes will die of starvation after being unable to integrate into a pack. Thus, the turtle nests available in the north of the island afford these local juveniles with improved food security and survival rates during this critical period. While this may be good news for dingoes, this is bad news for turtles. Marine turtles are large animals well protected by a hard carapace or shell; they also live in the ocean, and hence would not typically be considered at risk from a 16–17 kg terrestrial canid like dingoes (Behrendorff et al., 2016). However, turtles are vulnerable to dingo attacks when they come onto land to nest or bask, and observations of dingoes attacking adult turtles are not uncommon. We observed this on five separate occasions during our study, including one green turtle that was attacked while returning to the ocean, changing course on at least eight occasions and leaving a grim trail of blood in the sand (Figure 2). Haig (2003), Baker (2005) and Strydom (2004) also report dingoes attacking adult turtles while nesting on K'gari. Turtle eggs are particularly vulnerable to predation by a range of predators including feral pigs *Sus scrofa*, varanids, European red foxes *Vulpes*, bandicoots and also dingoes (e.g. King et al., 2023; O'Connor et al., 2017; Whiting et al., 2007). Marine turtles are generally careful where they nest and will return to the water if disturbed or unable to successfully dig a nest chamber. Continued harassment by predators or human disturbance can cause turtles to abandon nesting activity and, at times, causes them to jettison and ‘waste’ the entire clutch of eggs (McArthur, 2004) until they return to lay their next clutch 2 weeks later.

Dingoes are also known to consume substantial quantities of freshwater crocodile eggs (*Crocodylus johnsoni*) and olive ridley turtle (*Lepidochelys olivacea*) eggs in northern Australia. For example, Somaweera et al. (2011) reported that dingoes were the main predators of freshwater crocodile nests around Lake Argyle, while Chatto (2004) and Whiting et al. (2007) found that dingoes and other wild dogs were the highest source of egg mortality. For their capacity to reduce successful nesting, dingoes are a recognised Key Threatening Process effecting the conservation of marine turtles (Allen & Leung, 2012) and are often lethally controlled to prevent predation.

Given that a proportion of turtle nests on K'gari go unmonitored when they are laid away from the main rookery (Figure 1), we cannot reliably estimate the total number of nests consumed by dingoes. That nest predation rates were similar across different dingo pack areas (Table 2), however, suggests that the observed effects of dingoes on turtles in the north are likely to occur in other areas of the island as well. Dingoes from the Caree pack have also been observed waiting at a nest relocation cage for hatchlings to emerge, further reducing the survival rate of these marine turtle species of concern.

## 5 CONCLUSION

Our results reveal the previously unquantified importance of turtles to dingoes and dingoes to turtles on K'gari, which has significant local management implications. Some have speculated that extant dingoes have insufficient natural food resources or that bushfires on the island leave dingoes with insufficient natural food resources, exacerbating human‐wildlife conflict and leading to lobbying of management agencies to provide supplementary food subsidies to dingoes (Anon, 2015; Conroy, 2020; Elsworth, 2008; Hytten, 2009; Hoffman, 2010; Gunn, 2011; Kingston, 2020; Robson, 2011). Our data do not support this view, but rather suggest that the amount of food available to dingoes in the form of turtle eggs alone is sufficient to support northern K'gari dingo populations during the summer bushfire season in places where dingoes have access to turtle nests. This was likely the case in the 2020/2021 season, when a large wildfire burnt the northern half of the island during the study period (Meiklejohn et al., 2021; Queensland Government, 2021). Others have also claimed dingoes to be an inconsequential risk to turtles on the island. Our data do not support this view either, but rather suggest that dingo predation of turtle nests on K'gari may ultimately cause the collapse of the entire turtle rookery if left unmanaged. Given that K'gari turtles forage up to 3000 km away (Limpus, 1997) and marine turtles return to their birthplace to nest only after taking decades to reach sexual maturity (Greenwood et al., 2010; Miller, 2017), such a collapse caused by the continual loss of more than 80% of the seasonal egg and hatchling production (Department of Environment and Science, 2021) would effectively represent the expulsion from or local extinction of nesting marine turtles on the island, one of which is already critically endangered in the South Pacific (Limpus & Casale, 2015).

Given that dingoes cannot be removed in this case because of their own conservation and cultural value, managers grappling with this issue have few reassuring options. However, translocation of nests (Bradley & Strydom, 2020) and in situ protection of nests with mesh barriers (Nordberg et al., 2019; O'Connor et al., 2017) both appear to be successful turtle conservation strategies to at least avert predation of eggs before hatching. A range of interventions, including the seasonal closure of Ngkala Rocks to vehicular access, would further support a reduction in nesting turtle disturbance and hatchling survival. Though they commit management agencies to such actions indefinitely, we encourage the Queensland Government to prioritise discussions between co‐managers for the continuation and expansion of such activities as a means of ensuring the K'gari turtle rookery and Butchulla cultural connection are not lost forever. We also encourage continued monitoring of dingoes and other wildlife populations on the island as a means of identifying any species conservation problems before they arise. Such actions may be the best way of addressing these types of conservation conundrums and ensuring the most positive outcome for all species of conservation and cultural concern.

## AUTHOR CONTRIBUTIONS

**Linda Behrendorff:** Conceptualization (lead); data curation (lead); formal analysis (supporting); methodology (lead); writing – original draft (lead); writing – review and editing (equal). **Rachel King:** Formal analysis (equal); writing – original draft (supporting); writing – review and editing (supporting). **Benjamin L. Allen:** Formal analysis (equal); methodology (supporting); writing – original draft (supporting); writing – review and editing (supporting).

## CONFLICT OF INTEREST STATEMENT

The authors declare no conflict of interest.

## Supporting information


Table A1

Video S1

Data S1

## Data Availability

Data Accessibility: Provided as the Supplementary File.
