# Supplementary material for: Trouble in paradise: When two species of conservation and cultural value clash, causing a management conundrum
Source: Ecol Evol. 2023 Nov 16;13(11):e10726. doi: 10.1002/ece3.10726 (PMC10653987; doi:10.1002/ece3.10726)
Supplement: Supplementary file 1 — Table A1 [file ECE3-13-e10726-s003.docx]

Manuscript ID: ECE-2023-06-01019.R1

Manuscript title: Trouble in paradise: When two species of conservation and cultural value clash, causing a management conundrum

**Supplementary file**

Table A1: Frequencies of predated and non-predated nests across combinations of season (year) and pack

| Season | Pack | Not-predated | Predated | Total |
| --- | --- | --- | --- | --- |
| 2019 | Browns_&_Ngkala_packs | 2 | 14 | 16 |
|  | Rooneys_pack | 3 | 7 | 10 |
|  | Sandy_Cape_pack | 4 | 19 | 23 |
| 2020 | Browns_&_Ngkala_packs | 1 | 12 | 13 |
|  | Rooneys_pack | 4 | 30 | 34 |
|  | Sandy_Cape_pack | 14 | 64 | 74 |
| Total |  | 28 | 146 | 174 |
